# The effect of pandemics towards sustainable architectural evolution

**DOI:** 10.1007/s44213-023-00010-3

**Published:** 2023-05-31

**Authors:** Anastasia Evangelista Sumanti, Gabriela Emilly Xian, Didit Novianto

**Affiliations:** Department of Architecture, Institut Teknologi Sepuluh Nopember, Surabaya, Indonesia

**Keywords:** Architecture, Cities, Pandemic, Sustainability

## Abstract

The pandemic has hit the world since 165 CE, which has impacted on how the planners, architects, and authorities responded to its condition until this era. Reviews of some building typologies are conducted to grasp how the space designs react to some of the world’s most significant pandemics during human civilization. First, a literature study on the world’s deadliest pandemics was con- ducted and listed the pandemics with a death toll of more than 1 million. As a result, the period of pandemics before 800 CE was found to have influenced the development of buildings, architecture, and cities in Rome to the Mediterranean and emerged the typology of Valetudinarium. Then, Lazzarettos appeared during the 1300 to 1800 CE pandemic period as the impact of global trade. In com- parison, the pandemics after the 1800 CE period are predicted to impact West- ern Architecture’s popularity and early modernization in Asia. Furthermore, after several flu pandemics in the early 19th century, humanity has recently faced a significant pandemic. This study has two main findings. The first one, regarding architectural evolution in responding to pandemics from early plagues to Small- pox and polio, identified the response for quarantine facilities. Second, regarding the answer in this era of the pandemic, the sustainability concept can accommo- date and present as architecture. The idea covers the pillars of sustainability.

## Introduction

COVID-19 began to appear in 2019 and is still showing its existence to this day, resulting in more than 262 million population infected, reaching 5.2 million death tolls worldwide, and still in an uncertain situation since the virus developed new strains (World Health Organization, 2020). The COVID-19 pandemic also forces people to adopt the “New Normal” to avoid spreading the virus. At the same time, researchers and scholars in architecture are critically questioning the relation between the previous pandemic and architecture nowadays and finding the measures practiced before ensuring people’s health as a reflection of the recent crisis. Studies show that the built environment exhibited the ability to thrive after the pandemic (Chang V, 2020; Dejtiar F, 2020; Muggah R & Ermacora T, 2020). Infectious diseases also shaped the architecture, cities, and our built environments ((Gharipour M, 2021; Megahed & Ghoneim 2020).

The previous studies classified a direct and indirect link between architecture and the pandemic ((Foucault M, 2014; Lewis P et al., 2020; Novianto D et al., 2021; Novianto et al. 2021; Pevsner N, 1976). Several impactful pandemics indicated a direct human-to-human transmission, either through the air or through fluid and touch. Practically, with construction technology, the architects and planners applied isolated spaces to prevent more comprehensive information, which is closely related to partitions and walls, either permanent or temporary, that separate one room from another so that it will affect the physical characteristics of buildings.

Discussion and hypotheses on how the New Normal will shape our architecture, environment, and cities of the 2020s are also conducted. Furthermore, some lessons learned from pandemics recorded in history are discussed for consideration in planning a sustainable and resilient built environment.

Although several pandemics and epidemics were found in BCE, the historical evidence regarding the spread of infection and casualties still lacks proof. Moreover, the disease was always associated with the beliefs that spread fear (Foucault M, 2014; Gharipour M, 2021). A temple, Agora of Athens, was taken from the daughter of Leos, who was sacrificed to save the city from catastrophe (Ching F D K et al., 2017).

### Research method

In this paper, reviews act as a particular method of historical analysis. More than 270 pieces of information on pandemics and epidemics in the century are reviewed and highlighted on 20 deadliest world pandemics (killed more than 1 million). This research is needed to reflect on how the architects and planners changed the buildings/cities after the pandemic. Therefore, this study aims to review the architecture and history of city development many centuries ago by pointing out some existing building typologies. After that comparison to the current condition of COVID-19 pandemic is done to see the development done, the difference found, and design evolution added from past conditions with the current condition. Table 1. shows the pandemic list to understand how many pandemics humans has experienced. The pandemics and epidemics analyzed was also taken from this list after further investigation and considerations from previous literature studies and is further describe in part 2 about historical studies.

**Table 1:** Pandemic List

Time	Name	Death Toll	Population Lost
165-180	Antonine Plague	5-10 M	25-33% of Roman population
541-549	Justinian Plague	15-100 M	25-60% of European population
735-737	Japanese Smallpox	2 M	33% of Japanese population
1346-1353	Black Death	75-200 M	30-60% of European population
1519-1520	Mexico Smallpox	5-8 M	23-37% of population
1520-1527	New World Smallpox	56 M	Mexico, Central America, South America
1545-1548	1^st^ Cocoliztli Epidemic	5-15 M	27-80% of Mexican population
1576-1580	2^nd^ Cocoliztli Epidemic	2-2.5 M	50% of Mexican population
1629-1631	Italian Plague	1 M	Italy
1648-1905	Yellow Fever	150,000	Latin America and Africa
1656-1658	Naples Plague	1.25 M	Italy
1665-1666	Great Plague of London	100,000	London
1772-1773	Persian Plague	2 M	Persia
1817-1923	Cholera Pandemic 1-6	1 M	Worldwide
1855-1960	Third Plague	12 M	China, India, worldwide
1889-1890	Russian Flu (*H*_*2*_*N*_*2*_)	1 M	Worldwide
1918-1920	Spanish flu (*H*_*1*_*N*_*1*_)	40-50 M	1-5.4% global population
1918-1922	Russia Typhus	2-3 M	Russia
1957-1958	Asia Flu (*H*_*2*_*N*_*2*_)	1-4 M	Worldwide
1968-1969	Hong Kong Flu (*H*_*3*_*N*_*2*_)	1-4 M	Worldwide
1981-now	HIV	35 M	Worldwide
2002-2003	SARS	770	China, Asia
2009-2010	Swine Flu (*H*_*1*_*N*_*1*_)	284,000	Worldwide
2014-now	Ebola	11,000	Congo, Guinea
2015-now	MERS	850	Middle East
2019-now	COVID-19 (*SARS-CoV-2*)	5.2 M	Worldwide

## Historical reflection

### Valetudinarium in early plagues

**Fig. 1:**
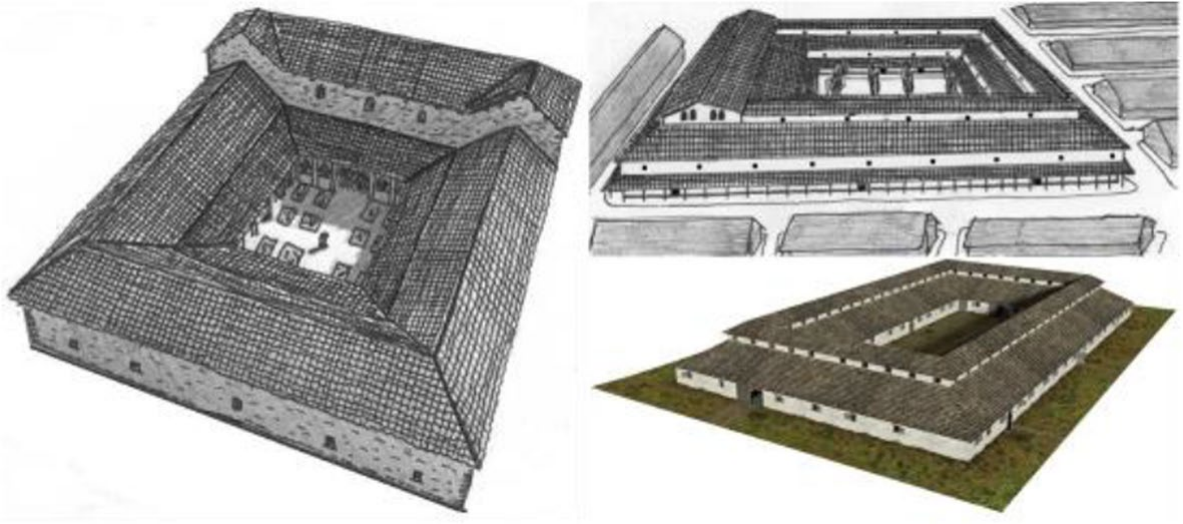
Valetudinarium Typology Source: Archeology, 2022 https://www.archeologiemusov.cz/en/educational-trails-and-museums/educational-trail-1/panel-8/

**Fig. 2:**
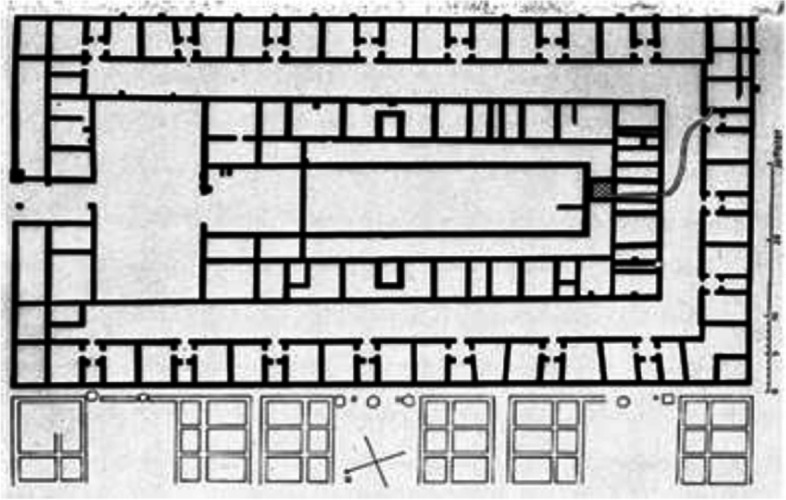
Valetudinarium Floor Plan Typology in Germany at Late 1st Century Source: Wikimedia, 2022 https://commons.wikimedia.org/wiki/File:Roman_Military_Hospital_-_Valetudinarium

During the early C.E., the first influential pandemic recorded was The Antonine Plague (165-180 CE), suspected to have been either Smallpox or measles (Furuse et al. 2010). Possibly brought to the Roman legion (today’s Italian peninsula) by armies that predicted 5-10 million deaths due to infections (Horgan J, 2019). The plague is believed to have first emerged during the Mesopotamian military forces of Romain 165 to 166 CE (Sicker M, 2000). At that time, healthcare facilities did not exist except the Valetudinarium (Figs. 1 and 2), which operated on military bases, serving the armies and enslaved people (Tiffany A et al., 2018). At that time, healthcare facilities did not exist except the Valetudinarium (Figs. 1 and 2), which operated on military bases, serving the armies and enslaved people (Tiffany A et al., 2018). The earliest Valetudinarium from Roman Empire was built in the First to Second Centuries when people needed more specific treatment than in private homes, which led to the development of temporary forts into permanent and became part of Roma’s fort architecture (Gabriel R A, 2012; McCallum & Jack Edward, 2008; Retief & Cilliers 2010). While its size varied, the number of wards was adjusted, and the capacity of each community was no more than three beds, so it was designed for maximum privacy (Cilliers & Retief 2002). Figure 2 shows that the typology consists of four buildings with rectangular forms connected by an entrance lobby. In addition, access is limited to a single entrance, which may prevent the disease from getting out of control and for security reasons. From the ground plan in Fig. 2, a broad and elongated corridor connects all the small rooms bounded by walls. Each building complex was constructed to accommodate 6-10% of the approximately 5,000 military troops (Gabriel R A, 2012). The building consists of an entrance hall, pharmacy, staff room, reception room, kitchen, and sanitary (Byrne E H, 1910). On the other hand, the earliest literary artifacts evidence about hospitals can be traced back to the 5th BCE (Arjuna Aluvihare 1993; Hope V, 2007; Risse G B, 1990; Söylemez M M, 2021). Endemic infections with diarrheal illnesses caused the Classic Maya’s collapse in the 8th century. This was then followed by the emergence of hospital typology, where patients are treated and isolated (Wright 1997).

### Lazzaretto in Bubonic Plague

During the Bubonic Plague period, it was dominated by Black Death; the deadliest recorded in human history emerged in Afro-Eurasia in 1346 and peaked in Europe in 1347 (DeLeo & Hinnebusch 2005). Black Death spread widely from the Mediterranean Basin to Africa, West Asia, and Europe (Sicker M, 2000). In addition, maritime trade between the Mediterranean and China, in the 13^th^ century, the Mongol occupation in Central Asia followed the trade flow, thus making it a decisive factor in spreading the plague. Also, in the 13th Century, Siena, one of the most important cities in Italy, came to an abrupt halt with the arrival of the Black Death, which reached Siena in 1348. Two-thirds of Siena’s one hundred thousand citizens had succumbed by the end of the year, and the city never recovered (Ching F D K et al., 2017).

On the other hand, in the 14th century, the monastic and feudal government systems shifted to mercantilism, decentralized governance and the loosening of the slavery system had a good effect on the recovery of the pandemic. Health services that were initially centralized and monopolized by the church became ineffective, prompting the establishment of public health facilities that adapted monastery architecture. The Bubonic Plague in 14th Century forced significant improvements in the urban Renaissance by clearing overcrowded residential housings, developing quarantine facilities, expanding the margins, and opening more common spaces (Megahed & Ghoneim 2020).

**Fig. 3:**
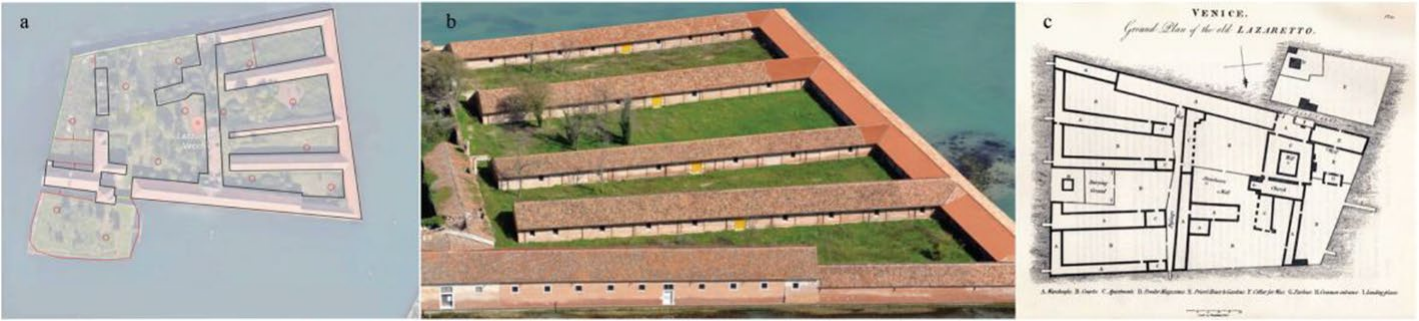
Lazaretto Vecchio Typology (**a**: site plan, **b**: aerial view, **c**: floor plan)Source: BG Architecture https://bg4fsvirginia.wordpress.com/2015/09/28/lazzaretto-nuovo-preliminary-research/

**Fig. 4:**
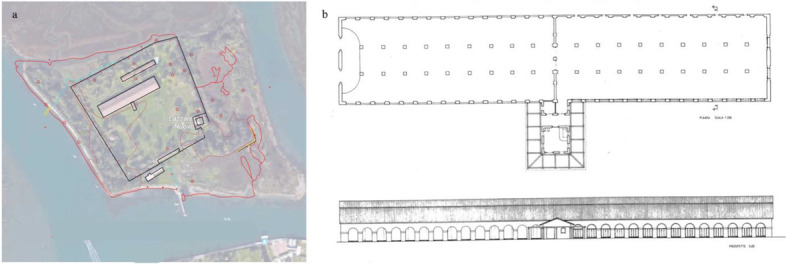
Lazaretto Nuovo Typology (**a**: site plan, **b**: floor plan) Source: BG Architecture https://bg4fsvirginia.wordpress.com/2015/09/28/lazzaretto-nuovo-preliminary-research/

Lazaretto was found on the Vigna Murata Island in 1468 based on the Senate of Serenissima decree to prevent the contagion during epidemics, named Nuovo (new) and Vecchio (old), operated between 1403 and 1630. Located close to the Lido, where clear plague cases were admitted, the island became a quarantine area for comers arriving from other ports suspected of being plague-infected. Lazarettes can be shipped permanently to anchors, isolated islands, or mainland buildings. In some Lazarettes, postal items were also disinfected, usually by fumigation. Figures 3 and 4 show the typology of Lazaretto Vecchio and Lazaretto Nuovo, respectively.

### Quarantine Facilities

In 1492, geological phenomena greatly influenced the climate, biosphere, and environment, including the encounter between Eurasian and American microbes. As many as 95% of the more than 20 million Americans died from exposure to the Eurasian disease, namely Smallpox, but only a few Europeans died from an American disease such as syphilis (Ching F D K et al., 2017). The trade routes between Eurasia and Africa and their close living habits to livestock make Eurasians much more resistant to disease than people in America. For example, the Black Plague has swept across Eurasia, killing nearly 10 percent of the population from China to Europe, which is undoubtedly an effect of global trade, but ultimately also boosts the immune systems of the survivors (Ching F D K et al., 2017). In the era of industrialization, infectious diseases such as cholera and typhoid impacted the sanitary reform movement, which contributed to the innovation of water and sewage systems to be straighter, smoother, and more expansive in response to pathogens (Ching F D K et al., 2017).

**Fig. 5:**
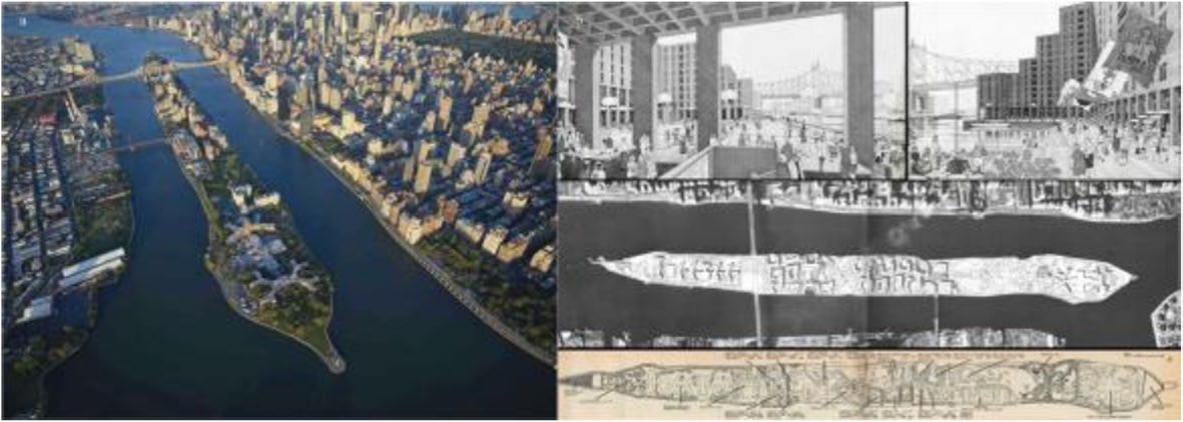
Roosevelt Island (a: aerial view, b: floor plan) Source: Tripsavvy, NYC Urbanism https://www.nycurbanism.com/brutalnyc/2017/2/15/eastwood

New York’s harbor has presented a unique case study for disease containment throughout many pandemics and disease outbreaks. Quarantine island becomes a phenomenon around the more important port of New York, serving various patients at different points in time, allowing for the isolation and treatment adjacent to the city’s density. Together with Blackwell Island and Welfare Island, Roosevelt Island (Fig. 5) is one of the most prominent of these islands due to its unique proximity to Manhattan and its development over time to produce efforts to quell the spread of the Smallpox 1856. The Smallpox Hospital was the first quarantine island hospital in the U.S. to receive residents who contracted the disease. In 1939 due to the Polio outbreaks, the Welfare Hospital for Chronic Disease was built, designed by architect Isadore Rosenfield, focusing on what was believed to be the buildings’ vital ability to provide curative forces of light and air.

### Sanatorium in respect to tuberculosis

**Fig. 6:**
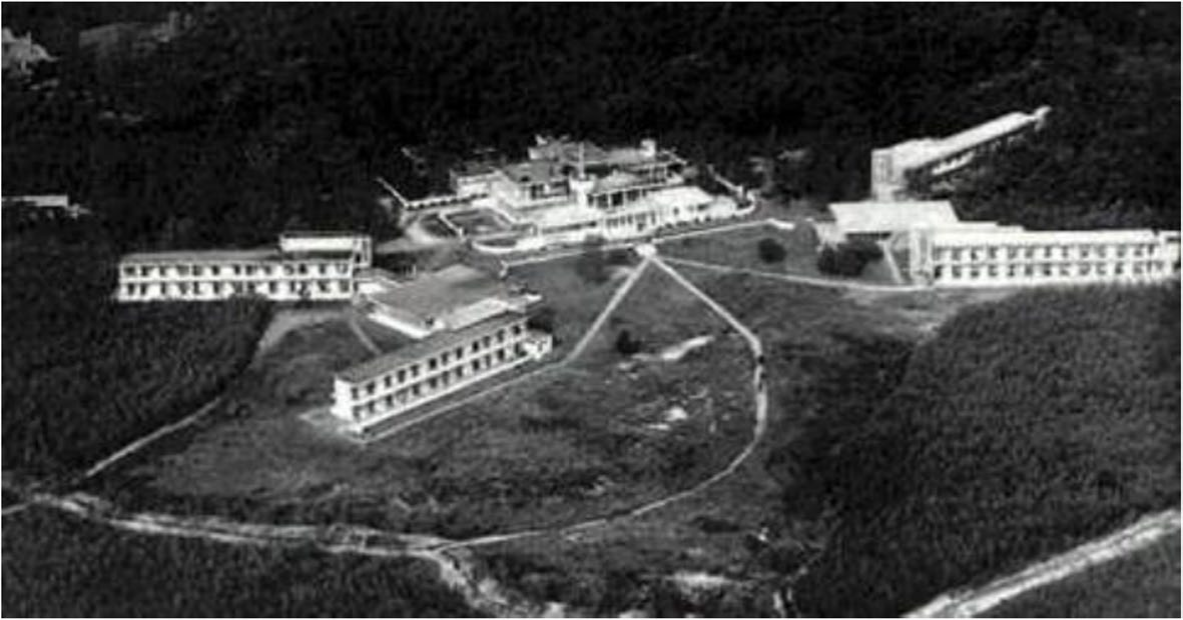
The Zonnestraal Campus of Sanatorium Source: Behance https://www.behance.net/gallery/106801773/Zonnestraal-Sanatorium

Tuberculosis is an infectious disease associated with poor working conditions. The outbreaks have persisted in modern times, characterized by their high contagiousness and often long recovery duration. Therefore, quarantining infected patients is a must in combating the disease spread. The isolation method began to place the patient in a Sanatorium (Fig. 6) in the late 19th century, a housing that provides healthy eating and outdoor living to those infected with Tuberculosis. Jan Duiker in 1931 built this new building typology focused on fresh air circulation, sunlight exposure, and immersion with nature.

## Flu, Covid-19, and sustainability

### Sustainability and pandemic

The modern period coincided with the westernization process in Southeast Asia, the development of the Neoclassical cities until early modernism, and finally, the development of contemporary cities in the era of globalization. At this time the flu disease type (HxNy) appeared in the early 19th century. During this period, some notable diseases also exist. Possibly, it occurred due to the rapid development of science so that new types of viruses are immediately detected and classified based on their family groups and characteristics. The other possibility is the influence of faster globalization which can no longer hinder the mobility of large numbers of people from one country/region to another. This is thought to have triggered the virus infection transmission a faster and more global impact than ever before. Infectious disease pushed urban renewal in the 20^th^ century. Here, architects and planners saw design as a solution for conditions like Tuberculosis, polio, and flu (Spanish), which influenced urban planning, tenement reform, slum clearance, and waste management (Chang V, 2020).

The COVID-19 virus has become the new age of pandemic design, building systems and conditions are affected by the virus infection spread manner which is air borne and can also be spread through animals, making areas with high density of population become red areas with high danger levels. The condition of COVID-19 also affects the human behavior and mindset about a place, considerations of outdoor spaces, sanitation, and stay period. Smooth surfaces are considered better because its easier to clean and there’s lesser moisture absorption making it much cleaner than surfaces with much harder ways to clean such as fabrics and small grids or pores. Areas that are outdoors are also more preferred because of the exposure to sun and UV making it a natural strategy of sanitary cleaning, in open spaces there are also considerations of air circulation, making airborne viruses not trapped in one room, making the used room easier to sanitize (Bereitschaft & Scheller 2020).

In this period of pandemic, many implemented a much more consumptive lifestyle and many tends to prioritize sanitation even if it’s much more destructive to the environment and is consumptive in a economic way, this can be seen from the extensive use of single used product in order to guarantee the cleanliness of a product especially those regarding respiratory systems and touch (Dharmaraj et al. 2021). But on the other hand, many also tries to work in a much more sustainable manner, such as construction, economic spending in a regional scale, personal spending in living, considering the high spike of economical degradation, many are done in areas of high infection number, because of the medical treatment urgency and limited resources.

Sustainability can be associated with the three pillars principle economic, environmental, and social (Purvis et al. 2019). These pillars can be explained further in detail with SDGs. Regarding the discussion of this pandemic study, sustainability goals can be used as a parameter. The sections chosen for this paper are Good Health and Well-being, Sustainable Cities, and Communities (Bappenas 2022). The COVID-19 pandemic has a big impact on public health, and other significant consequences, resulting in reducing economic activity, with a 3.0% recession currently being expected (Ozili & Arun 2020). This pandemic also has an influence on social and physiological aspect (Brooks et al. 2020; Nicola et al. 2020).

The COVID-19 cases in Brazil, shown that a high density, population, and lack of vegetation were contributing to a confirmed cases and deaths related to disease (Viezzer & Biondi 2021). The COVID-19 death cases also strongly correlated with the built environment. This phenomena is including the quality of housing and living conditions (Hui et al. 2020).

### Current design strategies in facing the Covid-19 pandemic

#### Public: instant health facilities

**Fig. 7:**
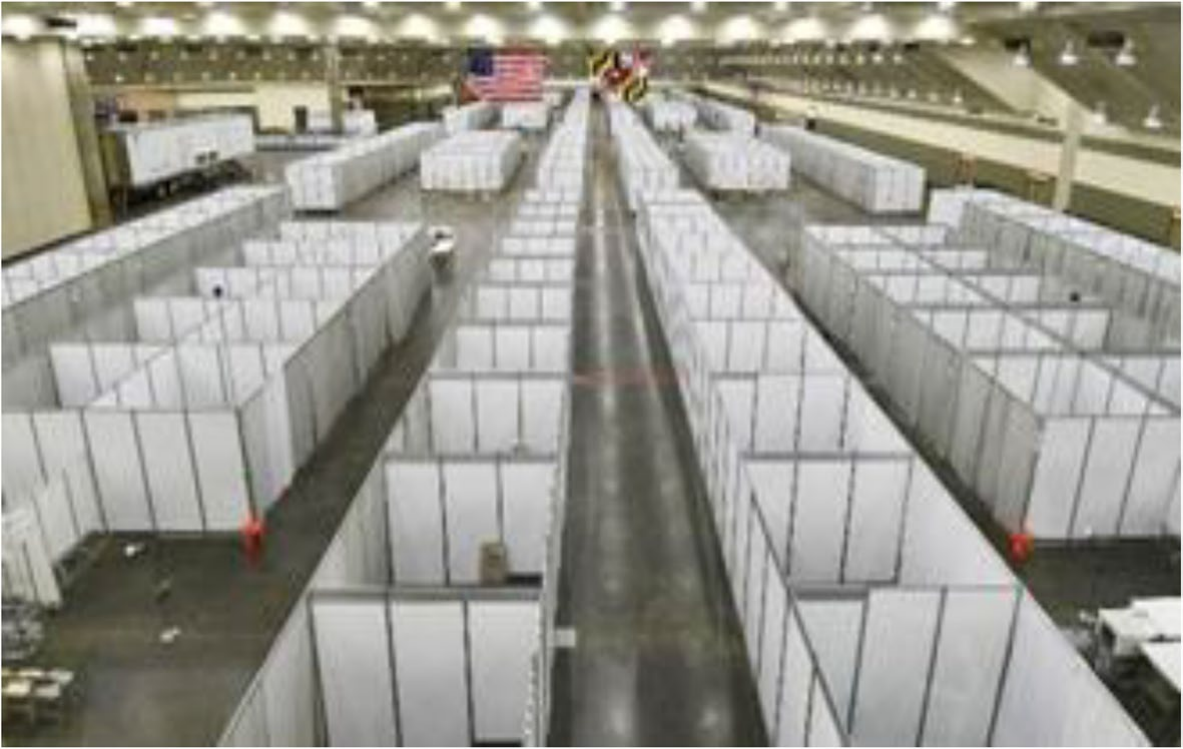
Convention Centers for Temporary Hospitals Source: USNews www.usnews.com/news/national-news/articles/2020-04-02/new-york-begins-construction

**Fig. 8:**
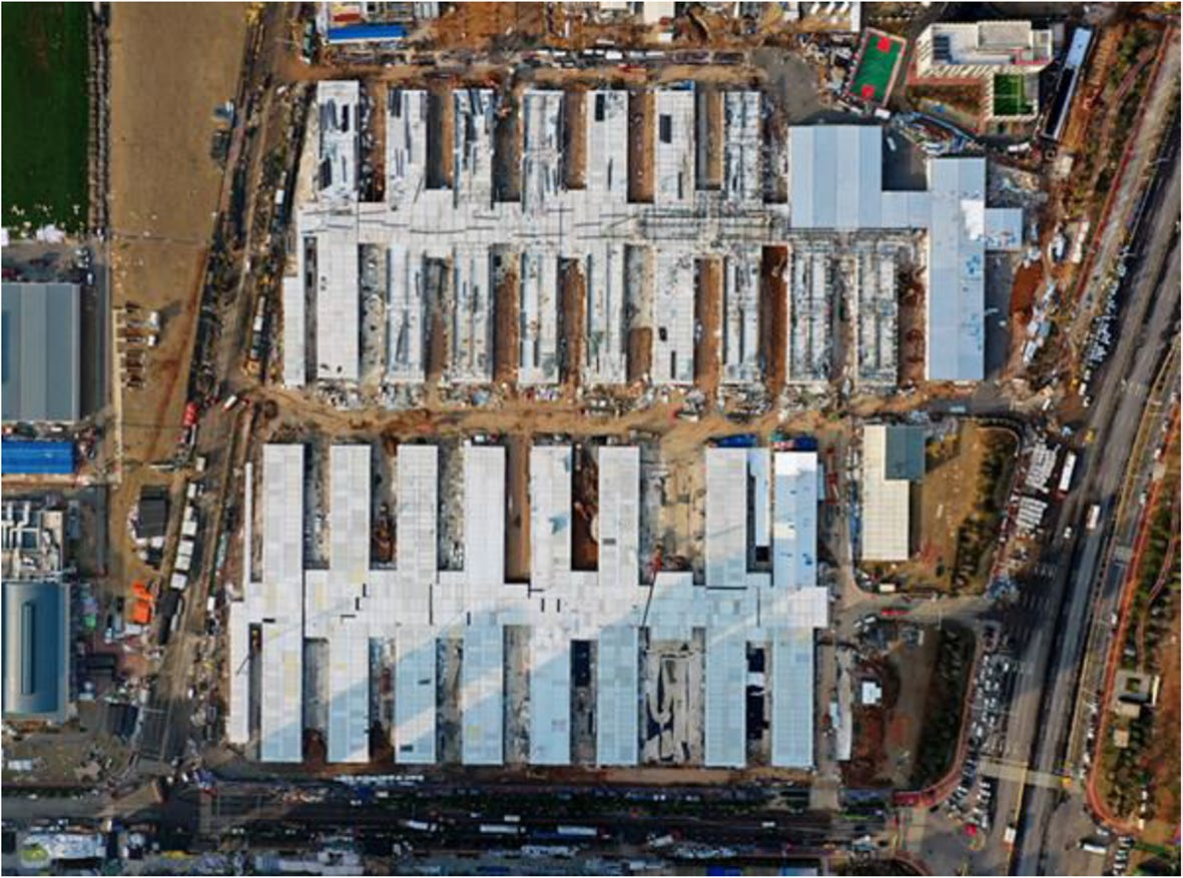
Top View of Leishenshan Hospital Source: The State Council the People’s Republic of China http://english.www.gov.cn/news/photos/202002/06/content_WS5e3b6d45c6d0a585c76ca866.html

At the start of the pandemic in Wuhan, the local government built a hospital with pre-fabrication materials, completed it in 6 days, and was designed to accommodate 1,000 beds, similar to the hospital in Beijing, which was created to respond to SARS in 2003 (Williams S, 2020). However, Fig. 7 shows convention centers were changed to temporary hospitals due to the existence of electrical, water, and sewage infrastructures (Serbu J, 2020). On the other hand, as an emergency response to the Covid-19 virus, Leishenshan Hospital uses a prefabricated construction combining prefabricated materials with modular construction (Fig. 8). This hospital can accommodate 1,500 beds located in 79,000 m^2^ (Wen-tao Li & Song-min Zhang, 2020). With the effective strategy of using unused buildings as emergency health facilities, it has proceeded to fulfill the SDGs goal of good health and well-being while also making a sustainable action in the aspects of economy and environment where it has saved energy consumption building cost from building a brand new one.

Sustainability in the context of the instant health facilities can be seen in the use of urgent strategies and its use of buildings and conventions that is most likely unused in the pandemic period where there's a limitation visitation or entrance, The temporary refunction can be very applicable in the context of minimum energy consumption, where there is no need for a new building be built from zero, making the energy that is supposably used for construction equals to zero.

#### Public: health protocol in buildings and cities

Screen and partitions have become common when the emergency status is lifted, and all office and commercial buildings are back to work. Masks and gloves are like the second skin, blocking transmission from many human activities. Emblems and warning signs to keep distance have become elements of architecture that cannot be avoided.

Limitations on the number of workers in a previously maximized room for maximum profit is also applied. In addition to avoid interactions between employees and customers, the circulation path of human activity is also made so that there is no face-to-face crossing between individuals. Restrictions on the number of workers are also intended to reduce the density of using public transportation. Public places also implement smartphone applications to record vaccination data and travel history as a requirement to enter a commercial building, such as malls and grocery stores.

On the other hand, extreme climatic and geographical situations for some regions are unavoidable. For example, cities in the four seasons experience extreme cold and heat climates. Meanwhile, tropical countries that experience hot weather throughout the year cannot be separated from air cooling conditioners. When energy/electricity is maximized to achieve people’s thermal comfort, Covid-19 has changed how people operate buildings. Keeping the air conditioner on, with air ventilation and windows open, has reduced the efficiency. The building’s electricity use has also increased significantly following the user’s comfort level, but this also depends on the economic status of the people. Most people with higher economic status tends to be much more consumptive and those with lower economic status tends to minimize spending because of the work limitation and lesser wage, affected by the pandemic condition and work hour cuts or job loss.

History also repeats when city parks, tourist beaches, and other public facilities are closed. But this time, not for sanitary reasons but to avoid the crowds in an area. In addition, in terms of city lifestyle and society, the impact of the pandemic on urban life is more pronounced when the business center has to walk by utilizing outdoor spaces such as roads and sidewalks so that the density of visitors that enters the more indoor areas with high viscosity and needs a certain period to open their face masks, such as cafes and restaurants can be moved or formed into a more open space.

The popularity of bicycles as a means of transportation in the city has also re-emerged, with health reasons to increase the body’s immune system through exercise. Covid regulations have also formed a brand-new standard of hygiene for every person in their daily life by implementing the hygienic routine of washing your hands more often, using sanitizing solutions, and using it to sanitize utensils or tables and public facilities. Along with that, cities that previously didn’t care about pollution and the environment have become unconsciously implemented sustainability from lifestyle changes since the Covid-19 pandemic because of the new habit made by the healthy covid lifestyle that medical professionals recommend to the public, such as more time with sports activities outside, a more nutritious diet with a more balanced diet fulfilling the purpose of Good Health and Wellbeing. Other than that, sustainability in the environment has also reached a new peak where a healthier lifestyle and less energy consumption are affected by the current post-pandemic lifestyle supporting the SDGs purpose of Sustainable Cities and Communities.

During the pandemic period self-quarantine that is done by most people especially those living in dense urban areas happen to affect the air quality in the city. Minimizing the use of private transportation, usage of working offices and communal areas, making pollutants decrease in concentration level (Sharifi & Khavarian-Garmsir 2020).

#### Non-Public: housing

COVID-19 also shows that the existence of health facilities is very crucial, where the progress of the infrastructure of a region or country greatly determines its resilience to sudden outbreaks. The COVID-19 pandemic has affected people’s appreciation of residential housing, and the living environment encourages house design that can accommodate self-quarantine or quarantine and protect from infectious diseases. Occupants become more aware of the lack of light in a room, air quality and ventilation, and the need for sanitation and hygiene. Spending a long time in a room will encourage residents to increase their comfort level and have a more flexible space with objects. It is also shown that even after the WFH (Work from Home) period, more people will use a house space to do their work activities.

Therefore, residential housing design might change significantly (Dejtiar F, 2020; Gao & Novianto 2018). Some studies have found a direct correlation between the crowd and health problems (Megahed & Ghoneim 2020; World Health Organization, 2020; Wright 1997). WHO recommended a healthy housing environment with surrounding green spaces, enhancing better infrastructure for social/physical distancing, and the maximum use of natural light, air, and other nature elements in detached housings. But right now, multi-story dwellings which have more shared spaces and utilities also need to focus on the touchless details for every shared area to give fundamental changes in this pandemic situation that transfers its sickness not only through the air that we breathe but also through touch, providing communal areas that can also keep its sanitary condition through a system is much needed in this pandemic period (Megahed & Ghoneim 2020).

The situation seems to have returned when there were no hospitals, so sick people could only wait for a doctor at home or return home for the poor after receiving treatment from a doctor. However, in contrast to that time, work can now be done from home. Therefore, from being an independent quarantine room, the house must also accommodate study and work activities, creating adaptable spaces for occupants to adapt to changing lifestyles and functions (Muggah R & Ermacora T, 2020). The need for a personal space that can be used for video-chat activities without background noise while maintaining privacy from other activities at home is also needed. In response, today’s residential buildings need to have large window openings that prevent noise, good balconies, and easy-to-clean surface materials to prevent the spread of disease, especially from viruses.

With the new housing condition and needs, the SDGs purpose of good health and well-being has been fulfilled on a much more micro and even individual level, where personal hygiene is much more considered. This condition can be said to be a sustainable behavior in social situations so that safety in health can also be sustained even in social conditions. It can also help in environmental sustainability, where natural lighting and air ventilation are more needed, making energy consumption much less than before the pandemic.

## Conclusion

When a deadly disease outbreak spreads rapidly and broadly to the scale of a pandemic, threats emerge to the structure and lives of communities. In the early period of civilization and the domestication of livestock since approximately 10,000 years ago, epidemics have claimed many victims. On a city scale, the spread of pandemics and epidemics has affected the distribution of supply logistics (often causing panic in communities) and social interactions.

The density of cities and the inability of health facilities to accommodate many patients force people to self-quarantine at home so that a particular room is needed in the house for isolation without transmitting the disease to other family members who are still healthy. Pandemics such as Smallpox, polio, and Tuberculosis require a recovery period of up to several months, so it is necessary to build permanent sanatoriums specifically designed to isolate the infected population or provide a healthier environment away from overcrowding. Several buildings, such as the Valetudinarium, Lazaretto, quarantine stations, open hospitals, Sanatoriums, and even the use of temples and public facilities, were deemed appropriate and became references to contemporary modern architecture. Throughout history, strategies in facing danger especially pandemic has evolved by analyzing the chances of how the infections spread and how to contain and heal the infected people in a local and authorized place. The periodical views and understanding of the environmental conditions also affect how generations react and approach on building the design strategies.

In the recent COVID-19 pandemic, not many architectural healthcare systems are built permanently or starting from zero, with sustainability and mindfulness in calculating time, economic conditions, the speed and urgency of the COVID-19 spread has also affected the approaches in facing the pandemic. much like the Valetudinarium, programs and functions changed, but other than efficiency and practicality, sustainability in the context of environmental and economic views are also considered, whether it is in the making of emergency public health care systems, housing strategies to decrease the number of chances in the spread of the COVID-19 virus transmission considering that it is an airborne virus that can easily spread through the air and through touch, and lastly by building a system that provides standardized scanning systems in public buildings on cities and urban areas. Modern people have increasingly touched an understanding of virus transmission both from the air, saliva, and others so that individually, the use of masks, face shields, and distance is done to protect oneself. Quarantine does not have to be carried out in hospitals but uses hotels, guesthouses, halls, and even private homes for mild symptoms.

Regarding this situation, it can be identified that the current pandemic: COVID-19, prioritizes the sanitary condition of the people on a personal and regional scale, but it can also be responded by architecture through sustainability, especially in Good Health and Well-being, Sustainable Cities, and Communities by building strategies and a new way of living, indirectly affecting the preferred design that’s suitable to the context period of COVID-19 pandemic. From the findings in this paper there are corrections that must be added to the equation of pandemic system and design. It is apparent that sanitation systems is needed and prioritized, but there are much better ways to stay clean but not wasteful or completely ignorant of the environmental or economic condition. Ways in the form of using personal reusable and disinfected tools instead of single use products, reusing and refunctioning buildings that is still in its prime in order to build an emergency health ward rather than building a new one from zero, avoiding mass over usage of energy and prioritizing looking and using a less energy consumptive lifestyle rather than personal comfort. in the future, it’s better to have a guideline and limitation on facing a new pandemic, even if it will take many years to meet a new massive and global pandemic such as COVID-19, but preparations of a more strategic and sustainable system can never be out dated or out of context.

## References

[CR1] Aluvihare A (1993). Rohal Kramaya Lovata Dhayadha Kale Sri Lankikayo Vidhusara Science Magazine.

[CR2] Bappenas (2022). Apa itu SDGs?.

[CR3] Bereitschaft, B., & Scheller, D. (2020). How Might the COVID-19 Pandemic Affect 21st Century Urban Design, Planning, and Development? Urban Science, 4(4). 10.3390/urbansci4040056

[CR4] Brooks, S. K., Webster, R. K., Smith, L. E., Woodland, L., Wessely, S., Greenberg, N., & Rubin, G. J. (2020). The psychological impact of quarantine and how to reduce it: rapid review of the evidence. In The Lancet. 395(10227). 10.1016/S0140-6736(20)30460-8 10.1016/S0140-6736(20)30460-8 PMC7158942 32112714

[CR5] Byrne EH (1910). Medicine in the Roman Army. Classical J.

[CR6] Chang V (2020). The post-pandemic style.

[CR7] Ching F D K, Jarzombek M, Prakash V. (2017). A Global History of Architecture (3rd Edition). Wiley, Inc. Hoboken, New Jersey.

[CR8] Cilliers, L., Retief, F. P. (2002). The evolution of the hospital from antiquity to the end of the middle ages. Curationis, 25(4). 10.4102/curationis.v25i4.806 10.4102/curationis.v25i4.806 14509111

[CR9] Dejtiar F (2020). Is coronavirus pandemic accelerating the digitalization and automation of cities?.

[CR10] DeLeo, F. R., & Hinnebusch, B. J. (2005). A plague upon the phagocytes. In Nature Medicine. 11(9). 10.1038/nm0905-927 10.1038/nm0905-927 16145573

[CR11] Dharmaraj, S., Ashokkumar, V., Hariharan, S., Manibharathi, A., Show, P. L., Chong, C. T., & Ngamcharussrivichai, C. (2021). The COVID-19 pandemic face mask waste: A blooming threat to the marine environment. In Chemosphere. 272. 10.1016/j.chemosphere.2021.129601 10.1016/j.chemosphere.2021.129601 PMC7836388 33497928

[CR12] Foucault M 2014 The politics of health in the eighteenth century J Foucault Stud ISSN 1832–5203 113 127

[CR13] Furuse, Y., Suzuki, A., Oshitani, H. (2010). Origin of measles virus: divergence from rinderpest virus between the 11 th and 12 th centuries. http://beast.bio.ed.ac.uk/Tracer. 10.1186/1743-422X-7-52 PMC2838858 20202190

[CR14] Gabriel R A. (2012). Man, and Wound in the Ancient World: A History of Military Medicine from Sumer to the Fall of Constantinople. Potomac Books.

[CR15] Gao, W, Novianto, D. (2018). Eco-house in Kitakyushu, Japan. In Sustainable Houses and Living in the Hot-Humid Climates of Asia. 483–490. Springer Singapore. 10.1007/978-981-10-8465-2_45

[CR16] Gharipour M. (2021). Health and Architecture: The History of Spaces of Healing and Care in the Pre-modern Era . Bloomsbury Publishing.

[CR17] Hope V. (2007). Death in Ancient Rome: A Sourcebook . Routledge.

[CR18] Horgan J. (2019). Antonine Plague, Ancient History Encyclopedia. World History Encyclopedia. World History Encyclopedia.

[CR19] Hui, D. S., I Azhar, E., Madani, T. A., Ntoumi, F., Kock, R., Dar, O., Ippolito, G., Mchugh, T. D., Memish, Z. A., Drosten, C., Zumla, A., & Petersen, E. (2020). The continuing 2019-nCoV epidemic threat of novel coronaviruses to global health — The latest 2019 novel coronavirus outbreak in Wuhan, China. In International Journal of Infectious Diseases. 91. 10.1016/j.ijid.2020.01.009 10.1016/j.ijid.2020.01.009 PMC7128332 31953166

[CR20] Lewis P, Nordenson G, Lewis J D, & Tsurumaki M. (2020). Manual of Physical Distancing-Space, Time, and Cities in the Era of Covid-19.

[CR21] McCallum, & Jack Edward. (2008). Military Medicine: From Ancient Times to the 21st century. ABC-CLIO .

[CR22] Megahed, N. A., & Ghoneim, E. M. (2020). Antivirus-built environment: Lessons learned from Covid-19 pandemic. Sustainable Cities and Society, 61. 10.1016/j.scs.2020.102350 10.1016/j.scs.2020.102350 PMC7313520 32834930

[CR23] Muggah R, Ermacora T (2020). Opinion: Redesigning the COVID-19 city.

[CR24] Nicola, M., Alsafi, Z., Sohrabi, C., Kerwan, A., Al-Jabir, A., Iosifidis, C., Agha, M., & Agha, R. (2020). The socio-economic implications of the coronavirus pandemic (COVID-19): A review. In International Journal of Surgery. 78. 10.1016/j.ijsu.2020.04.018 10.1016/j.ijsu.2020.04.018 PMC7162753 32305533

[CR25] Novianto, D., Hidayat, A. S., Hazrati, F. Y., Rahmavani, A. M., Fadhila, A. R., Jaya, A. M., Koerniawan, M. D., & Munawir. (2021a). Rethinking sustainability during WFH: A survey on living environment quality and energy use. IOP Conference Series: Earth and Environmental Science, 881(1). 10.1088/1755-1315/881/1/012005

[CR26] Novianto D, Nuffida N E, Gao W. (2021b). A Review of Architecture Response to the Pandemic towards New Normal Behavior. Int., Conf. and Workshop: Re-Thinking the HIBIKINO Campus 2001-2021b, 13–18.

[CR27] PK Ozili T Arun 2020 Spillover of COVID-19: Impact on the Global Economy SSRN Electron J 10.2139/ssrn.3562570

[CR28] Pevsner N 1976 A history of building types Thames and Hudson

[CR29] Purvis, B., Mao, Y., Robinson, D. (2019). Three pillars of sustainability: in search of conceptual origins. Sustainability Science, 14(3). 10.1007/s11625-018-0627-5

[CR30] Retief, F., Cilliers, L. (2010). The evolution of hospitals from antiquity to the Renaissance. Acta Theologica, 26(2). 10.4314/actat.v26i2.52575

[CR31] Risse GB 1990 Mending bodies, saving souls: a history of hospitals Cambridge University Press

[CR32] Serbu J. (2020). Army Corps sees convention centers as good option to build temporary hospitals. Federal News Network.

[CR33] Sharifi, A., Khavarian-Garmsir, A. R. (2020). The COVID-19 pandemic: Impacts on cities and major lessons for urban planning, design, and management. In Science of the Total Environment. 749. 10.1016/j.scitotenv.2020.142391 10.1016/j.scitotenv.2020.142391 PMC7499053 33370924

[CR34] Sicker M. (2000). The Struggle over the Euphrates Frontier. The Pre-Islamic Middle East Greenwood : Vol. ISBN 0-275-96890-1. Praeger.

[CR35] Söylemez MM (2021). The Gundeshapur School: Its History, Structure, and Functions. Am J Islamic Soc Sci.

[CR36] Tiffany A, Ziegler, & Troyanos. (2018). Medieval Healthcare and the Rise of Charitable Institutions . Springer International Publishing.

[CR37] Viezzer, J., & Biondi, D. (2021). The influence of urban, socio-economic, and eco-environmental aspects on COVID-19 cases, deaths and mortality: A multi-city case in the Atlantic Forest, Brazil. Sustainable Cities and Society, 69. 10.1016/j.scs.2021.102859 10.1016/j.scs.2021.102859 PMC7977034 33758745

[CR38] Wen-tao Li, Song-min Zhang. (2020). Modularization, Standardization and Prefabrication. Rapid Construction of Leishenshan Hospital.

[CR39] Williams S (2020). Coronavirus: How can China build a hospital so quickly?.

[CR40] World Health Organization. (2020). Coronavirus disease (COVID-2019) situation reports-Situation report-87.

[CR41] Wright, L. E. (1997). Intertooth patterns of hypoplasia expression: Implications for childhood health in the Classic Maya collapse. American Journal of Physical Anthropology, 102(2). 10.1002/(SICI)1096-8644(199702)102:2<233::AID-AJPA6>3.0.CO;2-Z. 10.1002/(SICI)1096-8644(199702)102:2<233::AID-AJPA6>3.0.CO;2-Z 9066902

